# Solvent Additive-Induced Deactivation of the Cu–ZnO(Al_2_O_3_)-Catalyzed γ-Butyrolactone Hydrogenolysis: A Rare Deactivation Process

**DOI:** 10.1021/acs.iecr.1c04080

**Published:** 2021-10-27

**Authors:** Vanessa Solsona, Silvia Morales-de la Rosa, Oreste De Luca, Harrie Jansma, Bart van der Linden, Petra Rudolf, José M. Campos-Martín, María Emma Borges, Ignacio Melián-Cabrera

**Affiliations:** † DelftChemTech, Faculty of Applied Sciences, Delft University of Technology, Julianalaan 136, 2628 BL Delft, The Netherlands; ‡ Sustainable Energy and Chemistry Group, Instituto de Catálisis y Petroleoquímica, CSIC, Marie Curie, 2 Cantoblanco, 28049 Madrid, Spain; § Zernike Institute for Advanced Materials, University of Groningen, Nijenborgh 4, 9747 AG Groningen, The Netherlands; ∥ Department of Chemical Engineering, Faculty of Applied Sciences, Delft University of Technology, Van der Maasweg 9, 2629 HZ Delft, The Netherlands; ⊥ Department of Chemical Engineering, School of Engineering and Technology, University of La Laguna, Avenida Astrofísico Francisco Sánchez, s/n, P.O. Box 456, 38200 San Cristóbal de La Laguna, S/C de Tenerife, Spain; # Applied Photochemistry and Materials for Energy Group, University of La Laguna, Avenida Astrofísico Francisco Sánchez, s/n, P.O. Box 456, 38200 San Cristóbal de La Laguna, S/C de Tenerife, Spain

## Abstract

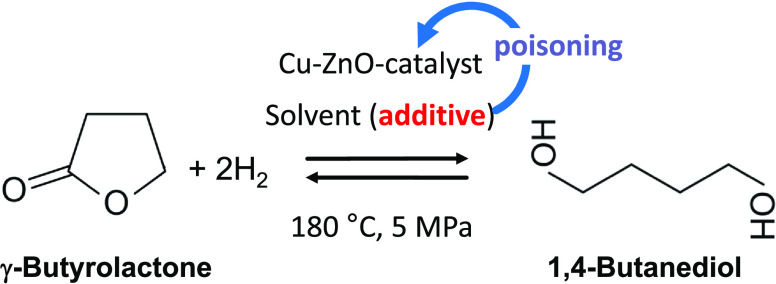

This work reports initial results on the effect of low concentrations (ppm level) of a stabilizing agent (2,6-di-*tert*-butyl-4-methylphenol, BHT) present in an off-the-shelf solvent on the catalyst performance for the hydrogenolysis of γ-butyrolactone over Cu–ZnO-based catalysts. Tetrahydrofuran (THF) was employed as an alternative solvent in the hydrogenolysis of γ-butyrolactone. It was found that the Cu–ZnO catalyst performance using a reference solvent (1,4-dioxane) was good, meaning that the equilibrium conversion was achieved in 240 min, while a zero conversion was found when employing tetrahydrofuran. The deactivation was studied in more detail, arriving at the preliminary conclusion that one phenomenon seems to play a role: the poisoning effect of a solvent additive present at the ppm level (BHT) that appears to inhibit the reaction completely over a Cu–ZnO catalyst. The BHT effect was also visible over a commercial Cu–ZnO–MgO–Al_2_O_3_ catalyst but less severe than that over the Cu–ZnO catalyst. Hence, the commercial catalyst is more tolerant to the solvent additive, probably due to the higher surface area. The study illustrates the importance of solvent choice and purification for applications such as three-phase-catalyzed reactions to achieve optimal performance.

## 1 Introduction

Catalyst stability and deactivation are fundamental properties that largely determine industrial applicability. In importance, stability can be considered comparable to selectivity. However, it tends to receive less attention than new selective synthesis routes or improved productivity. A high yield combined with stability is what makes a catalyst a case for further consideration in a development program.^1^ Heterogeneous catalysts can deactivate in a variety of manners such as poisoning (i.e., irreversible chemisorption), fouling or coking, thermal degradation (sintering or evaporation), mechanical strength-related phenomena, and corrosion-leaching under the reaction medium. There are in-depth studies on heterogeneous catalyst’s deactivation, among which to cite a few,^2−9^ mostly on gas-phase reactions.

Fundamentally, there exists a better understanding of gas–solid (two-phase) reactions than that of gas–liquid–solid reactions (also denoted as three-phase reactions). This can be justified by the higher complexity of the interaction between the liquid and the solid.^10−17^ That also means that deactivation studies for these reactions are scarce. Among the possible deactivation mechanisms in three-phase reactions, there exists an area that has been hardly documented: the effect of additives present in the solvent that may pass inadvertently for the experimenter.

In three-phase reactions, a solvent is used to dilute the produced heat from exothermal reactions. The solvent is usually employed in a large quantity. This means that if it contains an impurity, even at ppm level, the absolute amount can be comparable or higher than the concentration of active sites in the employed heterogeneous catalyst. A normal practice is to employ solvents without extensive additional treatment, though well-documented purification protocols exist.^18,19^ This study discusses the effect of low-concentration additives in commercial solvents. These additives are incorporated in the final formulation and are antioxidants that aim to prevent autoignition or autopolymerization during transportation and handling. The reason for their use is health and safety (H&S) for safe transportation from the production site to the final consumer. The bottom line is that such additives can have a negative impact on applications such as heterogeneous catalysis.

To illustrate this concept, we studied an industrial catalysis case study. 1,4-Butanediol (BDO) is a commodity of interest due to wide-ranging applications in the chemical industry. Most of BDO is produced by the multistage Reppe process.^20^ Alternatives to Reppe’s process have been proposed.^21−23^ Lately, a butane-based process was developed via maleic anhydride esterification. A more process-attractive route would be the direct hydrogenation of maleic anhydride, using noble metals, copper chromites, and Cu–ZnO catalysts.^24−27^

The original objective of this study was to investigate alternative solvents for the liquid-phase hydrogenation of γ-butyrolactone (GBL), which constitutes one of the steps in the maleic anhydride (MA) to 1,4-butanediol process via succinic anhydride (SA). The reaction is sketched in eq 1. Typically, 1,4-dioxane is used as a solvent; for simplicity, it will be denoted as “dioxane” from now onwards. However, dioxane has several disadvantages from the H&S point of view. The U.S. EPA classifies dioxane as “likely to be carcinogenic to humans” by all routes of exposure.^28^
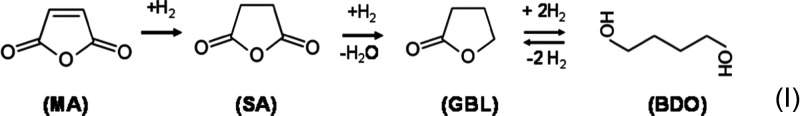
1To tackle this problem, we studied alternative solvents. Solvent selection for a three-phase reaction can be done by the inertness (i.e., weak adsorption on the active sites), safety, and price. Tetrahydrofuran (THF) fulfills better such requirements than dioxane. THF has a better H&S ranking as it has been classified as “suggestive evidence of carcinogenic potential”.^29^ In the CHEM21 solvent selection guide,^30^ dioxane was rated as “hazardous”, whereas THF scored better and was rated as “problematic”. The former means “the constraints on scale-up are very strong. The substitution of these solvents during process development is a priority”, whereas the latter (problematic) means “these solvents can be used in the lab or in the Kilolab, but their implementation in the pilot plant or at the production scale will require specific measures, or significant energy consumption”. Therefore, THF is a better option for replacing dioxane from the H&S perspective.

To assess THF’s suitability, both solvents were compared in the GBL hydrogenation using a Cu–ZnO catalyst; the latter has been claimed to be active and selective for this reaction.^24−26^ In the course of such study, we found surprising results: the negative effect of a stabilizing additive (2,6-di-*tert*-butyl-4-methylphenol) present in the commercial THF. Studying this phenomenon therefore became the scope of the present work. Preliminary insights are provided on this undocumented deactivation, which can also play a role in other applications where additives are present. The study was conducted on two catalytic systems, a lab-made binary CuO–ZnO and a commercial CuO–ZnO–MgO–Al_2_O_3_, upon reducing the Cu species.

## 2 Experimental Methods

### 2.1 Materials

The commercial CuO–ZnO–MgO–Al_2_O_3_ material was purchased from Alfa Aesar (ref number: 45776) and has a composition of CuO (60–68 wt %), ZnO (22–26 wt %), Al_2_O_3_ (8–12 wt %), and some MgO (1–3 wt %), according to the supplier. Table S1 describes the chemicals employed for the synthesis of the binary catalyst. Tables S2 and S3 list the chemicals employed for the catalytic testing of the binary Cu–ZnO and commercial catalysts, respectively. All employed gases were of high purity, >99.995 vol %. Note that the commercial catalyst, upon reduction for the reaction, is denoted as Cu–ZnO–MgO–Al_2_O_3_; however, XPS revealed the presence of unreduced Cu(II) species. For simplicity, the catalyst was denoted as Cu–ZnO–MgO–Al_2_O_3_ since the reduced Cu species are involved in the active sites.

### 2.2 Preparation of the Binary Catalyst

The binary CuO–ZnO oxide catalyst precursor was synthesized by coprecipitation at a constant pH of 7 (±0.1) using a tailor-made rig, using a similar procedure described by Melian-Cabrera et al.^31^ The process flow diagram can be found in Figure S1. A mixed aqueous solution of Cu(II) and Zn(II) nitrates and an aqueous solution of Na_2_CO_3_ (2 M) were added simultaneously, but at different rates, into a vessel containing 300 mL of distilled water at 70 °C. The concentration of the metal nitrates was ca. 1 M, whereas the individual concentrations of copper and zinc nitrates were adjusted to a copper molar fraction of 0.7 (Cu/(Cu + Zn)). The suspension was stirred and kept at pH ∼7 by automatically controlling the flow rate of the sodium carbonate solution, whereas the metal nitrate solution was pumped at a rate of ca. 2.5 mL/min using a peristaltic pump. The final slurry was aged under stirring at 70 °C for 1.5 h.

The solid was recovered by filtration and thoroughly washed with Milli-Q water to remove impurities such as sodium to be below 0.05 wt % in the calcined material. Finally, the solid was dried overnight at 90 °C and calcined in air at 350 °C for 6 h. The sample was labeled as Cu_70_Zn_30_, where 70 indicates the Cu mole percent and 30 is the Zn mole percent. The catalyst was activated before the reaction; the procedure is described in Sections 2.4.1 and 2.4.2. Figure S2 illustrates the steps for the synthesis and activation as a block diagram.

Two additional materials were prepared and used in this study for the X-ray diffraction (XRD) phase assignment of the ternary catalyst, namely, two oxide-based materials having a metal composition of Cu_5_Zn_95_ and Cu_50_Zn_50_. They were analyzed by XRD after ex situ calcination. The preparation was identical to the Cu_70_Zn_30_ described above.

The composition of the resulting oxidic materials after calcination was controlled by inductively coupled plasma optical emission spectrometry (ICP-OES) after digesting the solid in a HNO_3_ solution.

### 2.3 Catalyst Characterization

#### 2.3.1 Nitrogen Adsorption Measurements

The N_2_-adsorption measurements (−196 °C) were measured in a Quantachrome Autosorb-6B apparatus. Prior to the analysis, the samples were dried in vacuum at 200 °C for 16 h. The specific surface area was calculated by the Brunauer–Emmett–Teller (BET) method from the N_2_ adsorption isotherms. The total pore volume (*V*_T_) was determined from the desorption branch at a *P*/*P*_0_ ∼ 0.97. The pore size distributions were obtained using the Barrett–Joyner–Halenda (BJH) model. For all of these methods, the recommendations given elsewhere were followed.^32^ The texture of the commercial unreduced CuO–ZnO–MgO–Al_2_O_3_ catalyst was obtained in a Micromeritics ASAP 2420 equipment using a similar experimental protocol as described above.

#### 2.3.2 X-ray Diffraction

The powder XRD patterns were acquired in a Bruker-Nonius D-5005 diffractometer equipped with a graphite monochromator using a Cu Kα X-ray source. Data were collected in the range of 20–70 (2θ, degrees) with a step size of 0.02° and an accumulation time of 2 s (step mode). Identification of the crystalline phases was done using the JCPDS database. The Cu particle size was estimated by Scherrer’s equation (eq II)^33^
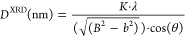
IIwhere *K* is a constant parameter that depends on the experimental conditions, λ is the wavelength of the incident X-ray (Cu Kα), θ is the angular position of the employed reflection, and the expression () is the corrected line broadening at half the maximum intensity; *B* is the line broadening at half the maximum intensity and *b* is a factor that corrects instrumental deviation on the order of ∼10^–6^ rad. This approach is used for Gaussian-type peak line shapes as in this case.^34^

The XRD patterns for the commercial Cu(O)–ZnO–MgO–Al_2_O_3_ catalysts were obtained in an X’Pert Pro PANalytical diffractometer equipped with a Cu Kα radiation source (λ = 0.15418 nm) and an X’Celerator detector based on real-time multiple strip (RTMS). The samples were ground and placed on a stainless steel plate. The diffraction patterns were recorded in steps over a range of Bragg angles (2θ) between 4 and 90° at a scanning rate of 0.04° per step and an accumulation time of 20 s. Diffractograms were analyzed with X’Pert HighScore Plus software.

#### 2.3.3 Determination of the Cu and ZnO Surface Areas

The copper surface area for relevant binary catalysts was measured by pulsed-N_2_O chemisorption at room temperature;^35^ this temperature avoids the oxidation of bulk Cu. Prior to analysis, the samples were reduced at 275 °C for 2 h in a flow of 20 vol % H_2_ in helium. After the reduction step, the gas mixture was changed into pure helium, and the sample was left at the same temperature for another 2 h to remove the physisorbed H_2_ before it was cooled down. During the chemisorption experiments, known amounts of N_2_O gas were pulsed into the carrier gas that flows through the sample bed. At the surface, metallic Cu was oxidized with the formation of Cu_2_O and the release of N_2_ that was quantified with a thermal conductivity detector (TCD). The chemisorption stoichiometry was assumed to be O/Cu_s_ = 0.5. The unreacted N_2_O was frozen out with a cold trap. The pulsing of N_2_O was continued until no N_2_ was released. The total amount of N_2_ was estimated by the cumulative area under the chromatographic peaks. The atomic surface density for Cu was assumed to be 1.46 × 10^19^ atoms/m^2^; this value was proposed by Evans et al.,^35^ assuming that the surface contains the three planes, (100), (110), and (111), equally present. The ZnO surface area was estimated, as an approximation, using the pulsed-N_2_O chemisorption data and assuming that the Cu/Zn ratio remains equal on the surface, which is a good assumption since these catalysts are bulky.

#### 2.3.4 Temperature-Programmed Reduction

The reductive activation of the binary catalyst was studied by temperature-programmed reduction (TPR). The experiment was performed in a tailor-made fixed-bed reactor (4 mm id) setup using pure gases (Figure S3). The reducing mixture (7.5 vol % H_2_ in Ar) was obtained by mixing pure gases with mass flow controllers. The temperature ranged from 25 up to 600 °C with a heating rate of 10 °C/min. Samples were diluted with SiC (80 mesh) to improve the heat transfer in the catalyst bed. Since the reduction profiles can be greatly perturbed by the experimental conditions, the operating variables were chosen in such a way that the line profile, peak position, peak resolution, and H_2_ consumption are measured accurately. The *P* parameter^36^ was employed as a criterion, which is an amplification of a previous one established by Monti and Baiker (parameter *K*),^37^ since it considers the heating rate. The quantity, given by eq III, should be as low as possible within the experimental sensitivity and, in any case, lower than 20 K. Initial experiments resulted in a high signal-to-noise ratio, and the resolution was insufficient to extract reliable information from the peak profiles. The experiments were then redone using the *P*-criteria
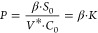
IIIwhere *S*_0_ is the initial amount of reducible oxide species (μmol); *C*_0_ is the initial hydrogen concentration (μmol/mL); V* is the total flow rate (mL/s), and β is the heating rate (K/s). Calibration of the produced H_2_ was done using a highly pure commercial CuO. The TPR pattern for the commercial CuO–ZnO–MgO–Al_2_O_3_ catalytic material was acquired in a Micromeritics TPR 2900 apparatus with a TCD detector using a similar experimental protocol as described above.

#### 2.3.5 X-ray Photoelectron Spectroscopy (XPS) Measurements

X-ray photoelectron spectroscopy (XPS) data were collected using a Surface Science SSX-100 ESCA instrument with a monochromatic Al Kα X-ray source (hυ = 1486.6 eV). The pressure in the measurement chamber was maintained at 1 × 10^–9^ mbar during data acquisition, while the electron take-off angle with respect to the surface normal was 37°. The analyzed spot had a diameter of 1000 μm, and the energy resolution was set to 1.26 eV for both the survey spectra and the detailed spectra of the Al 2s/Cu 3s, C 1s, Cu 2p, O 1s, and Zn 2p core level regions. Furthermore, an electron flood gun in optimized conditions was used during the XPS measurements to compensate for charging effects. Binding energies are referenced to the C 1s peak centered at a binding energy (BE) of 284.8 eV.^38^ All XPS spectra were analyzed using the least-squares curve-fitting program Winspec (developed in the LISE laboratory of the University of Namur, Belgium). Deconvolution of the spectra included a Shirley baseline subtraction and fitting with a minimum number of peaks consistent with the chemical structure of the sample, considering the experimental resolution. The profile of the peaks was taken as a convolution of Gaussian and Lorentzian functions; peak positions are reported ±0.1 eV when deduced from a fit. The uncertainty in the peak intensity determination is 2% for the Cu 2p, O 1s, and Zn 2p core level lines, 3% for the C 1s, and 4% for the Al 2s/Cu 3s core level regions.

The (Cu^0^ + Cu^+^)/(Cu + Zn + Al) XPS-derived parameter was employed as an alternative to pulsed-N_2_O data for the commercial catalyst interpretation. It is known that XPS cannot distinguish between Cu^0^ and Cu^+^ but for this particular case, this parameter can be a good approach to assess changes in surface Cu^0^. TPR revealed that CuO-like domains reduce into Cu^0^ at 240 °C. Since the fresh commercial catalyst was reduced at this temperature prior to the reaction, such species are in the Cu^0^ state. This was confirmed by XRD by the disappearance of the CuO and appearance of Cu^0^ in all spent catalysts. During the handling and analysis of the spent catalysts, it is possible that some Cu^0^ oxidizes into Cu^+^ at the surface, but this fraction will be equal for all of the spent catalysts. Therefore, it can be assumed that changes in the XPS contribution for Cu^0^ + Cu^+^ for the spent catalysts are due to changes in Cu^0^ and comparable to the use of N_2_O data. Having said that, the numerical values from the N_2_O data cannot be quantitatively compared to this parameter. Therefore, we compared values of this parameter for the commercial catalyst.

### 2.4 Catalytic Tests

#### 2.4.1 Binary Catalyst Testing

The hydrogenation experiments were carried out in a semibatch autoclave 500 mL reactor suite equipped with a gas-induced stirrer (Figure S4, Premex AG). A summary of the reaction conditions is given in Table S4. Above the reactor, an injection vessel was located to supply the reactant as soon as the reaction temperature was reached. The catalyst was first activated *ex situ* in a dedicated setup for pretreatment; it was reduced in a flow of 7 vol % H_2_/N_2_ (100 mL/min STP) at a heating rate of 5 °C/min to 240 °C for 2 h. The reduced catalyst was discharged into the stainless steel reactor containing 150 mL of the solvent; this delicate process was carried out within a protector glovebox (nitrogen pressure of 0.35 MPa) to avoid the oxidation of the catalyst by exposure to the ambient air. The activated catalyst was prevented from oxidation as it was surrounded by the solvent. The reactor was tightened and successively purged and vented with N_2_ and H_2_ at room temperature. The reactor was preheated under a hydrogen atmosphere (ca. 1 MPa) to the desired temperature. At that time, the γ-butyrolactone/THF mixture (5 mL γ-butyrolactone and 45 mL THF) was injected into the reactor through the reactant feeding system, after which the pressure was immediately adjusted to the experimental conditions, which corresponds to *t* = 0. The reactor was operated at a pressure of 5.0 MPa and 180 °C. The gas-induced stirrer was operated at a relatively high speed of 1500 rpm to guarantee a high gas–liquid interfacial area. At various time intervals, liquid samples were withdrawn from the reactor. These samples were analyzed offline using a Chrompack CP 9001 gas chromatograph equipped with a CP Sil8 CB column, a CP 9050 liquid sampler, and a flame-ionization detector (FID).

#### 2.4.2 Commercial Catalyst Testing

The commercial catalyst was tested in an equivalent reactor rig as described above and sketched in Figure S4, with the main differences being (1) the nominal size of the reactor was 1 L; for this reason, the amounts of reagents, solvents, and catalysts were increased to 450 mL of solvent, 11.25 mL of γ-butyrolactone, and 6.75 g of catalyst; (2) the catalyst was reduced *in situ* in the reactor before the reaction. The reduction of the catalyst consisted of placing the oxidic catalyst inside the reactor. The closed reactor was purged a few times with nitrogen to remove the atmospheric gas. Finally, the catalyst was reduced under a flow of 10 vol % H_2_/N_2_, by heating it from room temperature with a heating rate of 5 °C/min to 240 °C, and then keeping that temperature for 2 h. Then, the temperature was decreased to 180 °C, the system was pressurized with hydrogen, and then the reagent solution was gradually added using an injection vessel.

## 3 Results and Discussion

### 3.1 Model Catalyst

**Figure 1:**
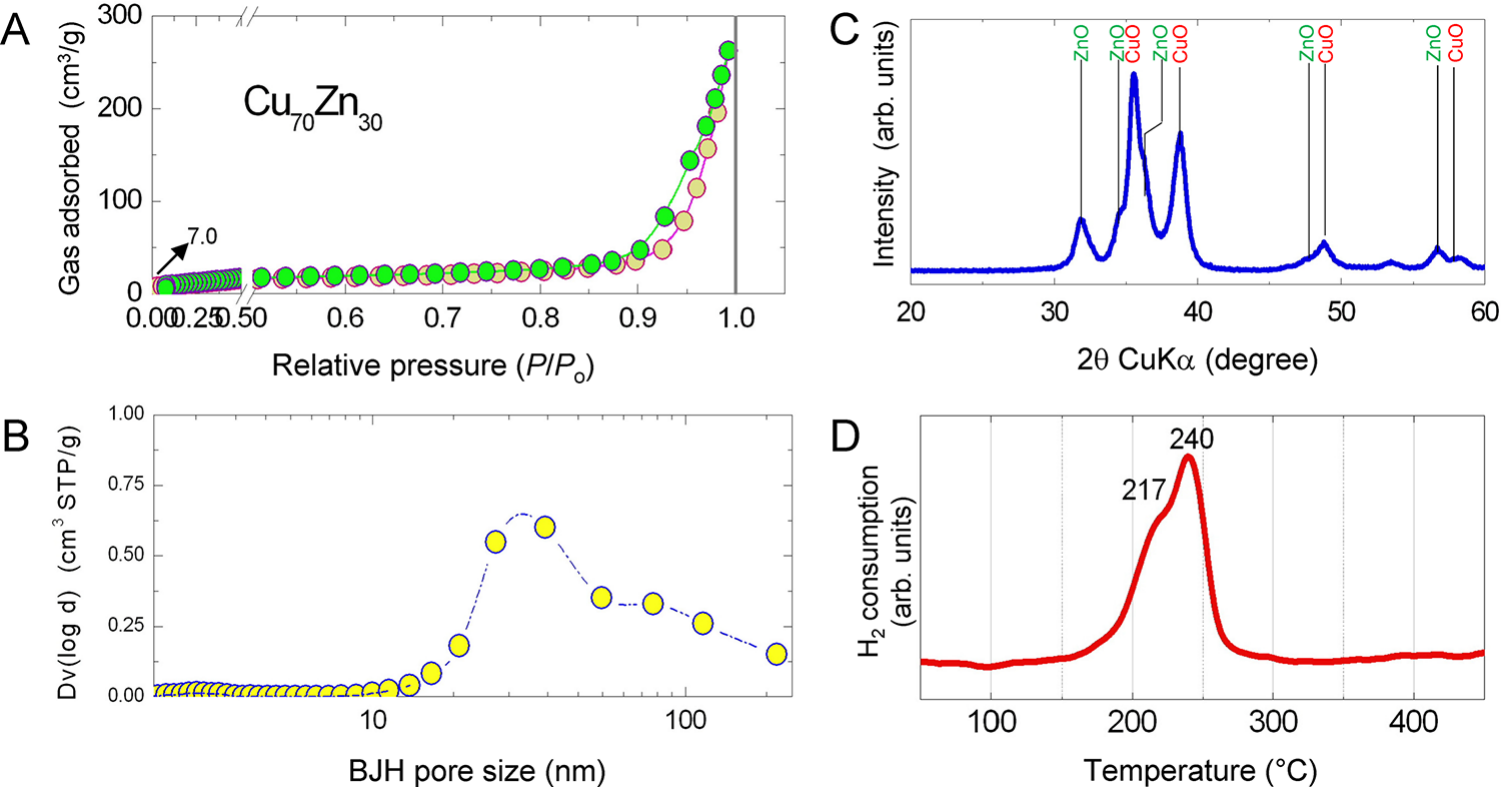
Characterization of the calcined Cu_70_Zn_30_ oxide catalyst precursor. (A) Nitrogen adsorption isotherms (−196 °C). (B) BJH pore size distribution derived from the N_2_ sorption data. (C) XRD diffraction pattern. (D) Temperature-programmed reduction.

The first tests were carried out on a lab-prepared model Cu–ZnO catalyst. Below, the main physicochemical features of the resulting material are described, showing that the material’s quality is acceptable before performing the catalytic tests. The CuO–ZnO catalyst precursor with a Cu/Zn mole ratio of 70:30 was prepared by coprecipitation. The dried hydroxycarbonate was calcined, and the oxidic material was characterized by chemical analysis, N_2_ physisorption, XRD, and TPR. The material displays a N_2_ physisorption isotherm having low-order meso- and macroporosities (Figure 1A). A BET surface area of 19 m^2^/g and a pore volume of 0.30 cm^3^/g were found. The BJH pore size distribution was located at ∼30 nm (Figure 1B) though the pattern indicates the existence of macropores as well (i.e., pores larger than 50 nm). The XRD profile (Figure 1C) shows CuO and ZnO as main phases; the relative intensity between the phases agrees with the Cu/Zn ratio. The TPR pattern reveals a reduction of the CuO starting at ∼160 °C and finishing at ∼270 °C (Figure 1D), with two maxima at 217 and 240 °C. Reduction of bulk ZnO occurs only at higher temperatures, but the baseline drift starting at 350 °C can be assigned to the start of the ZnO reduction (ZnO domains in interaction with Cu) and the decomposition of a residual carbonate phase.^39,40^ The quantification of the TPR profile reveals that all Cu(II) was reduced, with a H_2_/M molar ratio of 1.16. The higher value than unity is due to the partial reduction of ZnO; ZnO species in close interaction with Cu can form a Cu–Zn brass.^41^ TPR data also give a basis for selecting the reductive conditions to activate the CuO–ZnO prior to the reaction. Based on the TPR pattern, a temperature of 240 °C was chosen to activate the catalyst, which corresponds to the peak maximum of the second, and final, reduction step. Based on the above, the lab-prepared CuO–ZnO catalyst precursor (Cu_70_Zn_30_) shows good features and is appropriate to assess the solvent effect in the GBL hydrogenation.

**Figure 2:**
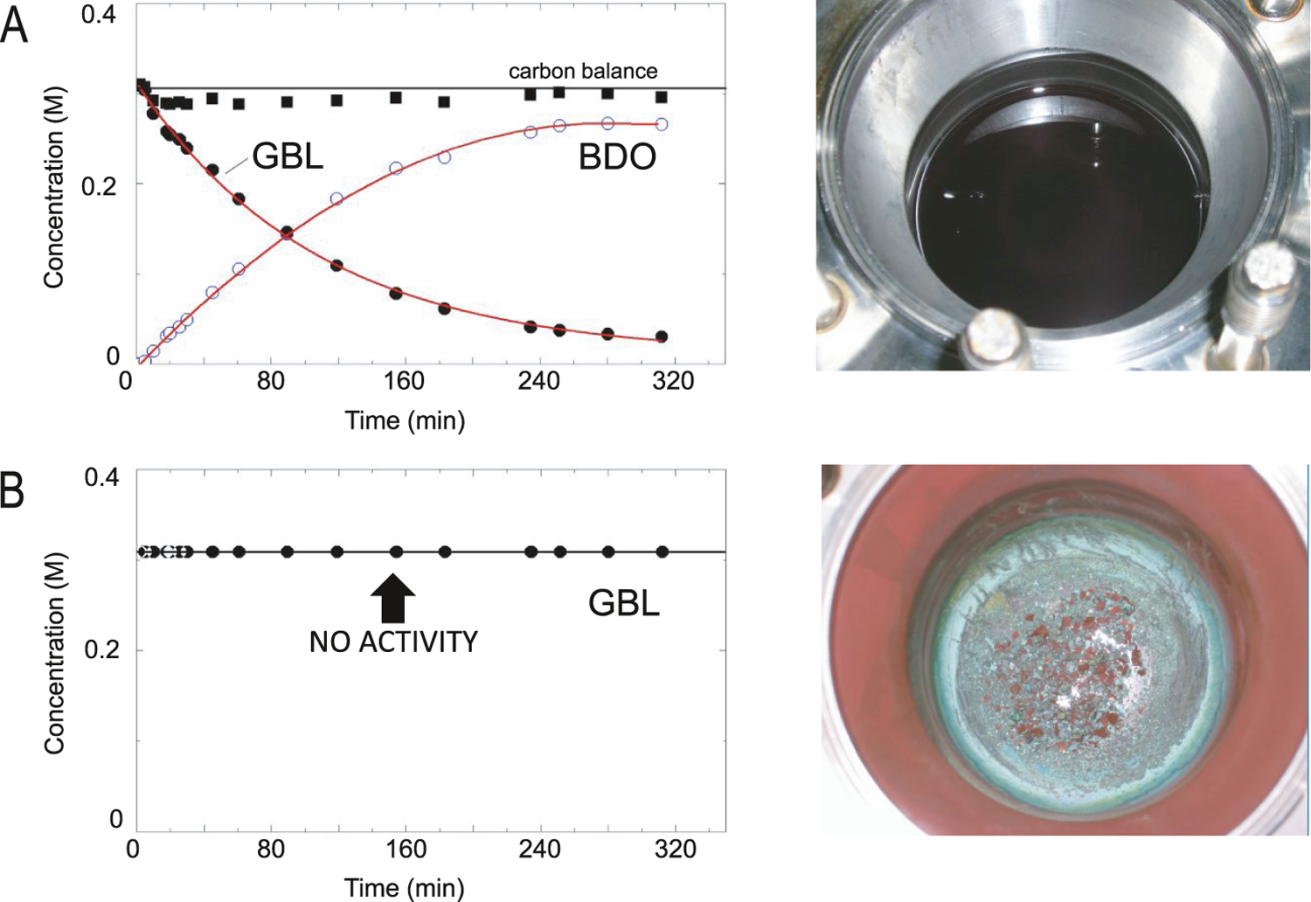
Binary Cu–ZnO catalyst performance. Hydrogenation of γ-butyrolactone over Cu–ZnO. (Left) Concentration profiles of the reactants and products as a function of time: γ-butyrolactone (filled circles), 1,4-butanediol (empty circles), and carbon balance (filled squares). Reaction conditions are given in Table S4. Solvents: (A) dioxane and (B) THF (type A). (Right) Appearance of the catalyst after reaction. The physical appearance of the catalyst under dioxane looked normal, a black-colored material. For the THF’s catalyst residue (THF, type A), the color indicated that the Cu catalyst might have sintered. The green-colored residue was unfamiliar to us. In panel (B, right), the solvent was removed and the catalyst was dried to better observe its appearance after reaction. GBL, γ-butyrolactone; BDO, 1,4-butanediol.

The performance of the Cu_70_Zn_30_ catalyst was evaluated in the GBL hydrogenation using dioxane as the solvent (Figure 2A). GBL was selectively converted (100% selectivity) into BDO. The plateau with an offset indicates that the reaction is equilibrium-limited. When dioxane was replaced by THF (a commercial THF containing the BHT additive, denoted as type A in this study), the outcome was completely different (Figure 2B). No activity was observed. While the physical appearance of the catalyst under dioxane looked normal (a black material), in the case of THF, the reddish color indicated that Cu in the catalyst might have sintered. The color may also come from polymeric residues, as claimed elsewhere;^27^ however, elemental analysis of the spent catalyst (using THF, type A) did not show differences as compared to that of a control case using dioxane, both ca. 3 wt % carbon.

Notably, the catalyst performance under this THF medium was catastrophic; a zero conversion was found. Experiments with this THF were repeated several times to be entirely sure that the effect was not just accidental; the negative outcome was proven reproducible. At that point, we looked at the specifications of the THF, in particular, at the additive 2,6-di-*tert*-butyl-4-methylphenol (BHT). This is a free-radical scavenger that inhibits explosive-prone peroxides by autoxidation. Without the stabilizer, peroxide formation can take place on storage or exposure to air or light. Despite it providing safety during transportation and handling, the effect of this additive in, e.g., a catalyzed reaction was unknown to date. This compound is acidic and can be adsorbed on the catalyst surface since ZnO is basic.

**Figure 3:**
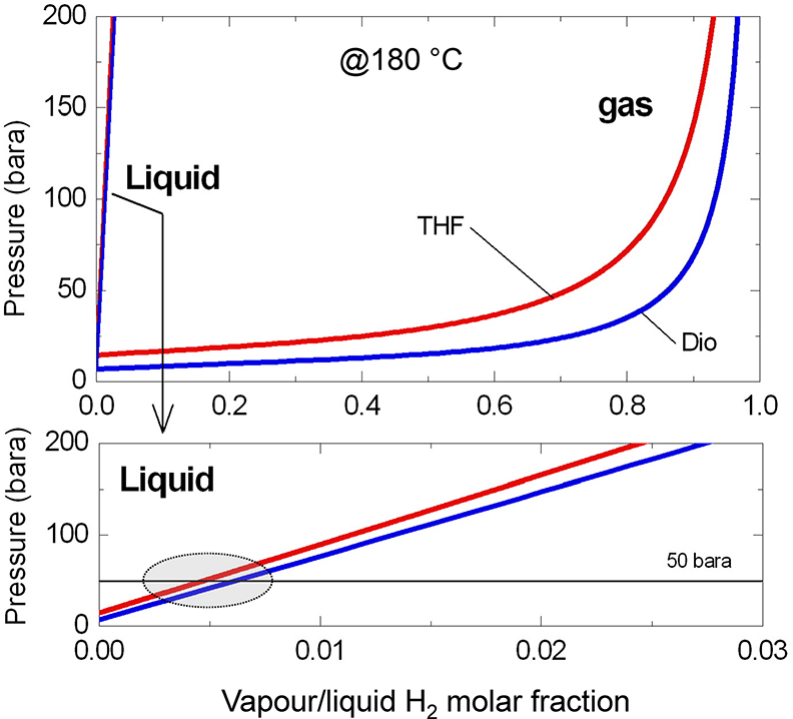
Hydrogen solubility in different solvents (THF, tetrahydrofuran; Dio, dioxane) as a function of pressure at 180 °C, calculated by the UNIQUAC^42^ contribution thermodynamic gas/liquid binary equilibrium method (Aspen Plus, Aspen Technology, Inc.).^43^

Preliminary characterization of the reduced (i.e., before reaction) and spent (i.e., after reaction) catalysts was done by XRD to understand the sintering phenomenon. An initial hypothesis was that the metallic Cu had sintered during the reaction. Calculation of the Cu-crystallite size using the XRD patterns showed an increase from 27 up to 47 nm (Table 1). Such an increase can be due to a few reasons. It can be related to differences in hydrogen solubility between dioxane and THF; a high hydrogen partial pressure would make sintering more remarkable. Calculations of the H_2_ solubility in the solvents at the reaction conditions by the UNIQUAC^42,43^ contribution method (Figure 3) did not show relevant differences between the solvents (highlighted area in Figure 3). Therefore, that hypothesis was ruled out. Another possible explanation is related to differences in viscosity among solvents. The viscosity of THF is 2.6 times smaller than dioxane (room-temperature data; Table S5). Hence, the shear force cannot explain the Cu agglomeration.

**Table 1:** Structural and Chemisorption Properties of the Cu–ZnO Catalysts

catalyst	properties	Cu^XRD^ (nm)^a^	S_Cu_ (m^2^/g)^b^	Cu^N_2_O^ (nm)^c^
Cu_70_Zn_30_	reduced	27	8.4	4.2
Cu_70_Zn_30_	reduced, after reaction, THF type A	47	5.9	6.0
Cu_70_Zn_30_	reduced, after reaction, dioxane	32	7.3	4.9

a The XRD patterns can be found in Figure S5.

b Values determined by room-temperature N_2_O chemisorption. The samples were previously H_2_-reduced at 275 °C for 2 h. Chemisorption stoichiometry O/Cu_s_ = 0.5 and atomic surface density of Cu = 1.46 × 10^19^ atoms/m^2^.

c N_2_O chemisorption-derived data. Average equivalent Cu-crystallite size (in nm) according to (600·*X*_Cu_)/(8.92·*S*_Cu_), where *X*_Cu_ is the copper mass fraction and *S*_Cu_ is the copper surface area determined by N_2_O chemisorption.

**Figure 4:**
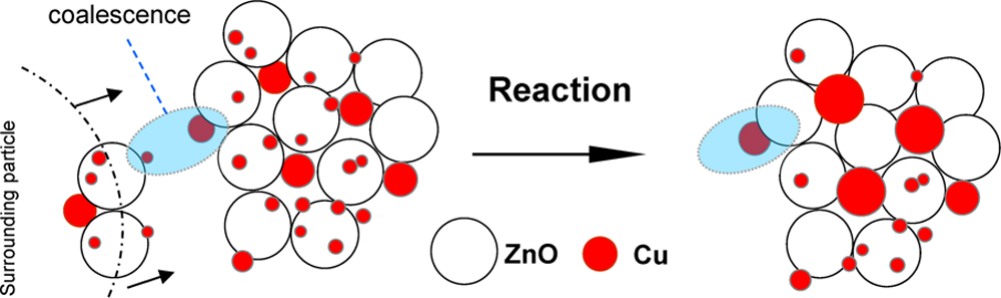
Proposed mechanically induced sintering mechanism under turbulent stirring conditions for a Cu–ZnO catalyst during the liquid-phase hydrogenolysis of GBL under THF as solvent. GBL, γ-butyrolactone; THF, tetrahydrofuran.

A possible interpretation can be found in differences of mechanical stress, the stress of the suspended solid under mechanical stirring. The Hüttig temperature for Cu is ca. 178 °C, while that for ZnO is 476 °C (the Hüttig temperature is calculated as 1/3 × *T*_m_, where *T*_m_ is the melting temperature in K).^44^ The Hüttig point is the temperature at which the surface of the solids becomes mobile, facilitating metal surface diffusion. At the reaction temperature, 180 °C, i.e. above the Cu’s Hüttig point, it is plausible that the Cu crystallites become mobile; smaller ones can merge with bigger ones by collisions between the catalyst particles. A second aspect to be looked at is the system’s turbulency. A comparison between the Reynolds impeller numbers (*Re*_I_) between dioxane and THF was done in eqs IV and V
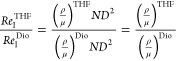
IVwhere *N* is the stirring speed, *D* is the impeller diameter, ρ is the density, and μ is the viscosity. After applying the corresponding data (Table S5), it leads to
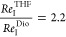
VThis means that the fluid-dynamic regime when employing THF is more turbulent than in dioxane, and the collisions between the suspended catalyst particles are more intense (i.e., more collisions per time leading to side effects as explained next). It is proposed that the particle collisions during the reaction, under THF, in combination with the low Hüttig temperature for Cu might provoke small Cu clusters to merge with larger ones, leading to bigger crystallites. This hypothesis is illustrated in Figure 4. In dioxane, such a difference in Cu particle size was not observed by XRD (Table 1); it slightly increased from 27 (reduced) up to 32 nm (spent in dioxane). Hence, the solvent fluid-dynamic properties appear to be related to such a sintering effect.

However, a more important descriptor for this reaction is the exposed metallic Cu that can be determined by pulsed-N_2_O chemisorption. This parameter gives information about the small Cu particles, which contribute most to the metallic surface area and therefore to the reaction’s activity. Pulsed-N_2_O chemisorption is a method employed to assess the exposed metallic surface area, which is a good measure of the activity of Cu-based catalysts,^31,35^ though there can be other descriptors. The freshly reduced catalyst showed a surface area of 8.4 m^2^ Cu^0^/g (Table 1). That would correspond to particles of ca. 4.2 nm on average. The chemisorption result for the THF-spent catalyst (type A) resulted in 5.9 m^2^ Cu^0^/g with an effective particle size of 6 nm, which means that the particle size increased by ca. 40%, while according to the XRD, it increased by ca. 75%. On the other hand, a particle size of 4.9 nm was found for the dioxane-spent catalyst; both N_2_O chemisorption and XRD show a similar particle size increase of ca. 17–19% when using dioxane. Overall, the chemisorption-derived particle sizes range narrowly between 4.2 and 6.0 nm. The N_2_O-derived Cu^0^ particle size for the THF experiment, compared to dioxane, is not as large as to expect a total deactivation. Combining the XRD and pulsed-N_2_O data, two phenomena seem to occur with the additive-containing THF: (1) a sintering effect yielding larger Cu particles (detected by XRD), while (2) the smaller Cu particles (pulsed-N_2_O) do not vary much between the solvents. As the small Cu particles are a better descriptor for the reaction than XRD, since they contribute most to the metallic Cu surface area, the only manner to explain the total deactivation is BHT poisoning. A preliminary explanation is given next.

**Figure 5:**
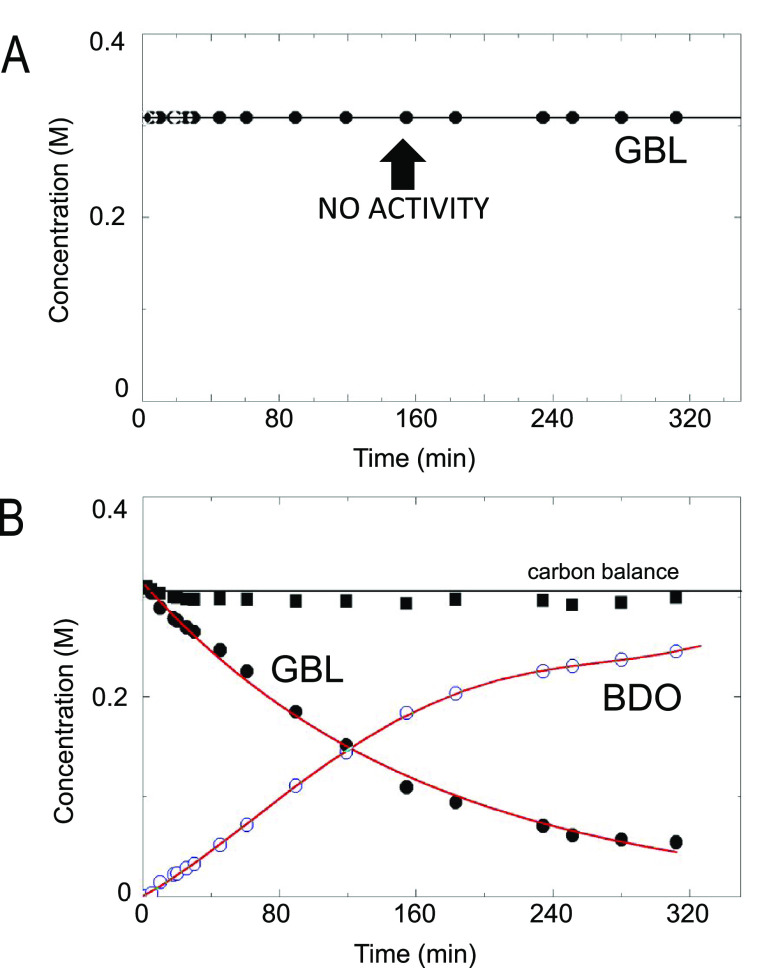
Binary Cu–ZnO catalyst performance. Hydrogenation of γ-butyrolactone over Cu–ZnO. Concentration profiles of the reactants and products as a function of time: γ-butyrolactone (filled circles), 1,4-butanediol (empty circles), and carbon balance (filled squares). Reaction conditions are given in Table S4. Solvents: (A) BHT-containing THF (type A) and (B) BHT-free THF (type B). GBL, γ-butyrolactone; BDO, 1,4-butanediol; and BHT, 2,6-di-*tert*-butyl-4-methylphenol.

A control experiment was done using BHT-free THF as solvent (denoted as type B in this study). Under this ultrapure THF, the catalyst was active though somewhat slower than dioxane (Figure 5). Therefore, the reason for the total deactivation under BHT-containing THF can be preliminarily ascribed to the presence of BHT that acts as a poison.

**Figure 6:**
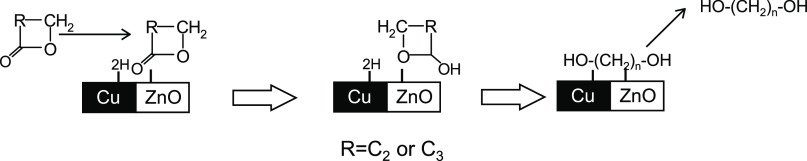
Active site model and reaction mechanism for the ring-opening hydrogenolysis of γ-butyrolactone to 1,4-butanediol over Cu–ZnO. Adapted with permission from Hamminga et al.^45^

The effect may be initially rationalized considering the reaction mechanism and the structure of the active sites. Hamminga et al.^45^ proposed for this reaction that the ZnO serves as the basic adsorption site where the lactone is first adsorbed, whereas the metallic Cu enables the dissociation of molecular H_2_; the ring-opening hydrogenolysis takes place, and the diol is released (Figure 6). Since BHT is an acidic compound, it can be expected that it adsorbs on the ZnO or Cu–ZnO interfaces and therefore blocks the reaction from going forward. The active site model requires therefore the presence of Cu–ZnO interfaces. It is known that these interfaces are available in limited quantity due to the bulky nature, or low surface area, of these catalysts (among others, Nakamura et al.^46^). Therefore, as the absolute quantity of the sites is low, these catalysts are expected to be prone to poisoning. A calculation was made to evaluate if the poisoning hypothesis can be considered solid. The total amount of BHT in a batch experiment is ∼200 μmol. From the pulsed-N_2_O chemisorption data, the amount of exposed ZnO can be calculated (see the calculation in the Supporting Information), resulting in ∼262 μmol of surface ZnO present in the catalyst for a batch experiment. Due to the bulky catalyst nature, it can be said that the Cu–ZnO interface is smaller in quantity than 262 μmol. Therefore, BHT can quantitatively poison the interfacial Cu–ZnO sites and the ring-opening hydrogenolysis does not proceed as it should occur in a poison-free Cu–ZnO site.

Another aspect is that water can hydrate/oxidize the active site with a negative impact. This has been discussed for Pd/Al_2_O_3_-based hydrogenation catalysts.^47^ To the best of our knowledge, the employed commercial solvents and reagents were of dried quality. Therefore, that effect will not be discussed further.

### 3.2 Commercial Catalyst

**Figure 7:**
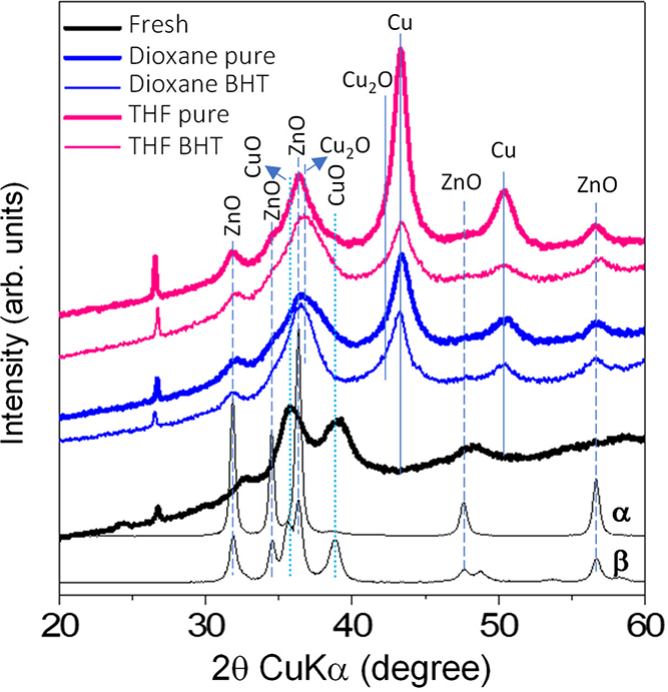
XRD diffraction patterns for the commercial Cu(O)–ZnO–MgO–Al_2_O_3_ catalysts, including the as-received oxidic material (fresh) and the after-reaction catalysts. The patterns of two CuO–ZnO reference compounds were included to help in the identification since the commercial catalyst contains broad reflections: (α) Cu_5_Zn_95_ and (β) Cu_50_Zn_50_, where the subscripts represent the relative mole composition. Both were prepared using the method described in Section 2.2.

In a final study, we tried to understand the effect of the solvent additive when using a commercial Cu-based catalyst. For this, we employed a Cu-based catalyst containing 60–68 wt % CuO, while the rest is ZnO, Al_2_O_3_, and in minor proportion MgO. The experimentally determined BET area is 137 m^2^/g (unreduced catalyst), a factor of ∼7 with respect to the binary CuO–ZnO catalyst precursor (19 m^2^/g). The H_2_-TPR profile shows a broad decomposition centered at 240 °C (Figure S6), which is comparable to the binary catalyst, as discussed earlier due to CuO reduction. The XRD pattern displays broad reflections of CuO and ZnO phases (Figure 7), whereas no phases were observed for Al_2_O_3_ and MgO. For the XRD phase identification, the use of two reference materials was required, in addition to the JCPDS files, due to the broad peaks.

**Figure 8:**
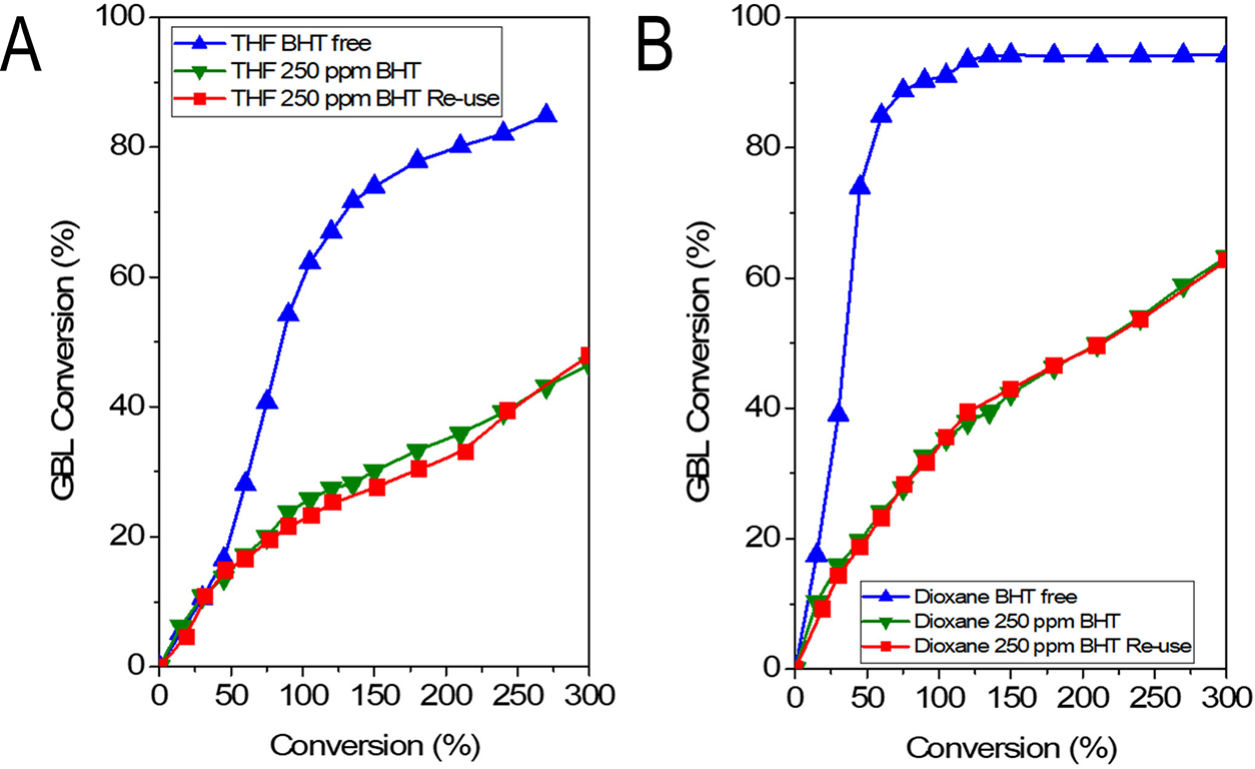
Performance of a commercial Cu–ZnO–MgO–Al_2_O_3_ catalyst in the hydrogenation of γ-butyrolactone using (A) THF as a solvent, with and without BHT as an additive, and (B) using 1,4-dioxane as a solvent, with and without BHT as additive. For THF, BHT comes as an additive in one of the commercial grades, whereas for 1,4-dioxane, the BHT was added by us to reach the same concentration, 250 ppm. For the experiments containing BHT, the catalyst was reused in a second cycle using fresh reagents after *in situ* reduction. The concentration profiles can be found in Figure S7. GBL, γ-butyrolactone; THF, tetrahydrofuran; BHT, 2,6-di-*tert*-butyl-4-methylphenol.

**Figure 9:**
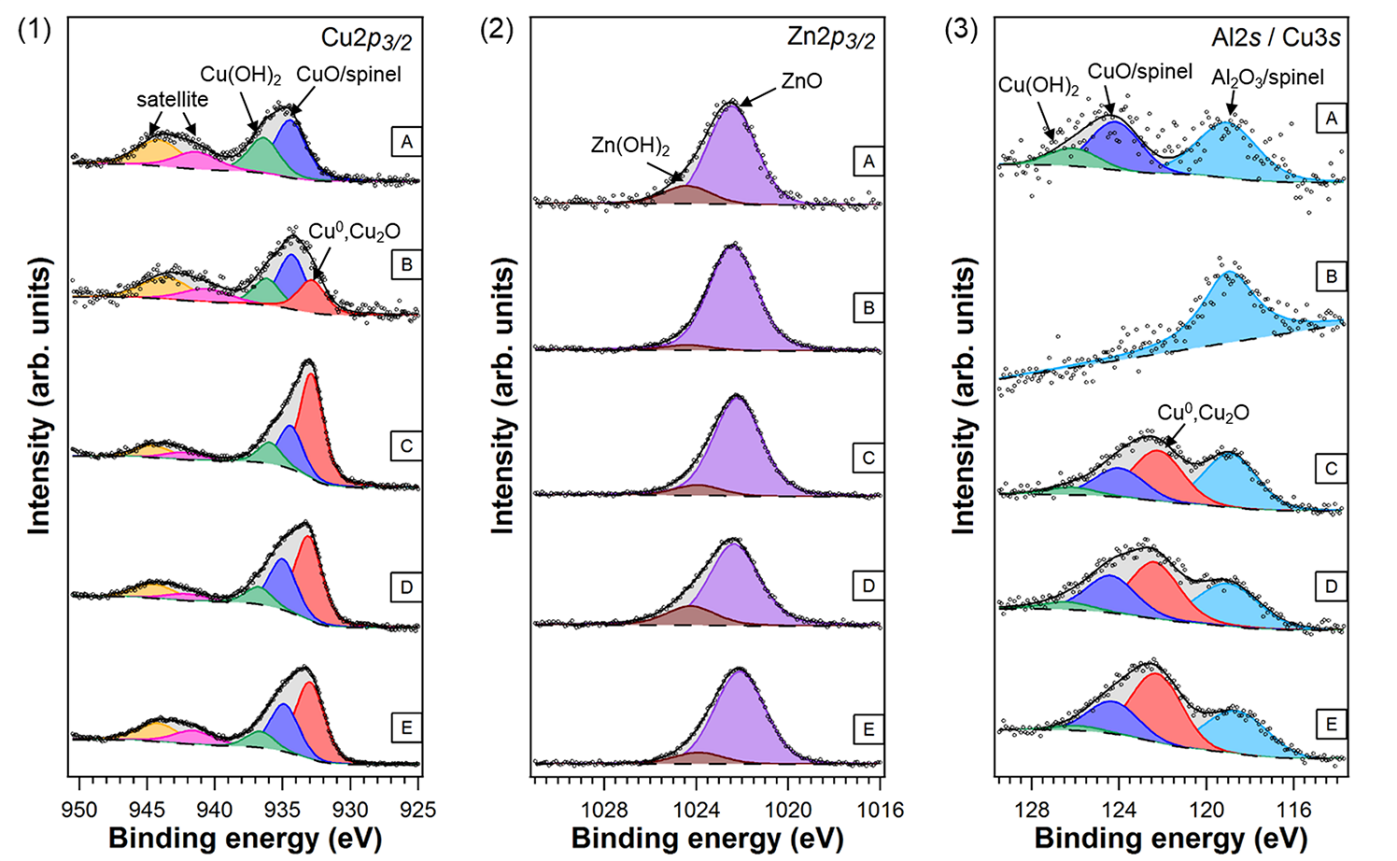
XPS spectra of the (1) Cu 2p_3/2_, (2) Zn 2p_3/2_, and (3) Al 2s core level regions for the various Cu(O)–ZnO–MgO–Al_2_O_3_ materials: (A) fresh commercial unreduced catalyst; (B) after-reaction, pure THF; (C) after-reaction, pure dioxane; (D) after-reaction, BHT-containing THF, and (E) after-reaction, BHT-containing dioxane. THF, tetrahydrofuran; BHT, 2,6-di-*tert*-butyl-4-methylphenol.

The reaction was carried out using the same solvents, THF and dioxane. THF was used in two commercial grades, with BHT and an ultrapure type without BHT. Pure dioxane was employed as received, as well as by adding a certain amount of BHT mimicking the concentration in THF type A, i.e., with 250 ppm BHT. In all cases, the selectivity was 100% with BDO as the sole reaction product (see the concentration profiles in Figure S7); therefore, the catalyst performance was assessed in terms of conversion. The performance using both pure solvents (Figure 8A,B) was acceptable; the conversion increases with reaction time toward achieving an equilibrium plateau. The performance with pure dioxane was faster than with pure THF. It seems that the active sites are suffering a sort of deactivation when using pure THF. Perhaps, it is the same effect as that observed in the binary system due to mechanically induced sintering (Figure 4). XRD patterns of the spent catalysts (Figure 7) show a sharpening of the Cu^0^ reflections with pure THF. Quantification of the XRD-particle size indicates, however, a moderate increase from 6.3 nm (dioxane) to 7.7 nm (THF type B) (Table S6). The study requires further insights with additional characterization (e.g. XPS or pulsed-N_2_O) to assess sintering on the smallest Cu particles. For this, an XPS study was carried out, where Cu 2p_3/2_, Zn 2p_3/2_, and Al 2s core level spectra were evaluated (see Figure 9). From those spectra, the atomic ratio of (Cu^0^ + Cu^+^)/(Cu + Zn + Al) in the probed volume can be determined. This parameter can be used as an approximation to the N_2_O data (see the explanation in Section 2). XPS spectra of the Mg LMM Auger line, O 1s, and C 1s core level regions before and after reaction treatments are shown in Figure S8 (see the Supporting Information).

The detailed Cu 2p_3/2_ spectra of all samples require three components to obtain a good fit (see Figure 9(1)). The peak at a BE of 933.0 eV (marked in red in Figure 9(1)) is ascribed to Cu^0^/Cu^+^ species, while the component at 934.4–935.0 eV (blue) corresponds to Cu^2+^ species.^48,49^ The additional peak at higher binding energy (green) can be assigned to copper hydroxide (Cu(OH)_2_).^49^ Furthermore, the shake-up features appearing at higher BEs (marked in fuchsia and orange in Figure 9(1)) are evidence for the presence of Cu^2+^ species in all samples.^50,51^ As the spent catalysts were reduced at 240 °C, the presence of CuO-like species in those catalysts can be attributed to a CuAl_2_O_4_ spinel that has a similar binding energy to CuO.^52^ The CuAl_2_O_4_ spinel reduces at a high temperature of ca. 430 °C.^53^ This spinel cannot easily be detected in the XRD patterns (Figure 7) since the peaks are quite broad in the region where the spinel should appear.

The detailed Zn 2p_3/2_ spectra for all samples, shown in Figure 9(2), require two components to obtain a good fit. The main peak at a BE of about 1022.2 eV (marked in purple in Figure 9(2)) corresponds to ZnO species, while the small component at higher BE is assigned to Zn(OH)_2_ species.^54^

Figure 9(3) presents the Al 2s/Cu 3s core level region. The component located at a BE of ∼119.0 eV corresponds to the Al_2_O_3_ species for the fresh sample (A); the same component can also be ascribed to the formation of CuAl_2_O_4_ species (the Al_2_O_3_ and CuAl_2_O_4_ species cannot be resolved as distinct components).^52^ The features at higher BEs in Figure 9(3) are the components of the Cu 3s peak, which correspond, respectively, to the chemical species already shown in the detailed Cu 2p_3/2_ spectra (see Figure 9(1)).

The (Cu^0^ + Cu^+^)/(Cu + Zn + Al) parameter for the pure THF catalyst is much lower than that for pure dioxane (3% vs 21%; see Table 2). This means that sintering of the small Cu particles occurs and can explain the slower rate under pure THF.

**Table 2:** XPS-Derived Surface Properties of the Fresh and Spent Cu(O)–ZnO–MgO–Al_2_O_3_ Catalysts

catalyst	properties	a^,^^b^
commercial	fresh, oxidic phases	0
commercial	spent, pure THF	3
commercial	spent, pure dioxane	21
commercial	spent, THF containing 250 ppm BHT	22
commercial	spent, dioxane containing 250 ppm BHT	26

a This parameter was used as an alternative to the pulsed-N_2_O data. See Section 2 for more details.

b Mg could not be quantified because the Mg 2s core level region overlaps with the Zn 3p core level. Other lines such as Mg 2p and Mg 1s displayed a high signal-to noise ratio. Therefore, it was assumed that Mg is present in low concentration at the surface.

The reaction with the BHT-containing solvents revealed a significant depletion of the conversion (Figure 8) for both cases. A second cycle revealed the effect to be reproducible. Note that between the reaction runs, the catalyst was reduced; therefore, the adsorbed BHT on the catalyst may have been eliminated during the thermal treatment of reduction under a flow of 10% H_2_ in nitrogen. This explains the same trend in the second run. In other words, it is a chemical inhibition of the sites during the reaction without affecting the Cu crystallites. There are smaller conversion levels with BHT-containing THF than with BHT-containing dioxane. The reason may be related to the suggested mechanically induced sintering (Figure 4), which also occurs when BHT is present. In fact, the (Cu^0^ + Cu^+^)/(Cu + Zn + Al) ratio for BHT-containing THF was 22%, while it amounted to 26% for BHT-containing dioxane (see Table 2); therefore, Cu sintering seems to occur as well in the BHT-containing THF but less than in pure THF. The reason can be associated with a possible stabilizing role of BHT, which binds more strongly the Cu particles and reduces sintering. These are preliminary observations using the XPS data primarily showing trends. Further insights using pulsed-N_2_O chemisorption data would be useful as it provides specific information of the Cu^0^. Moreover, the role of Zn may also be considered as it has been claimed as having a role in the active site for methanol synthesis.^40,46,55−57^

In general, in the catalytic tests, there is a less severe effect of BHT for the commercial catalyst, as compared to the binary counterpart; the binary one showed no conversion at all, whereas the commercial one showed a depletion. The better behavior may be related to the higher surface area of the commercial catalyst, a factor 7. In other words, its higher surface area tolerates better the 250 ppm BHT concentration as there are more active sites available; some are poisoned, while others are still free for the reaction. The XRD patterns of the spent catalysts (with BHT) show similarity (Figure 7), displaying Cu and ZnO as main reflections. There seems to be some Cu_2_O overlapping the other reflections. This phase arises from the surface oxidation of the metallic Cu after exposure to air during the manipulation and analysis (this effect is also visible for the pure solvent-derived materials). The metallic Cu reflections for both catalysts (with BHT) are very similar, as well as the particle size quantification, 5.8 nm vs 6.5 nm (Table S6). An important observation from XPS is that the (Cu^0^ + Cu^+^)/(Cu + Zn + Al) ratio remains high for the BHT-containing experiments and close to the pure dioxane experiment (see Table 2). This means that there are enough surface Cu species for the reaction to occur at a higher rate. The explanation for the depletion in the performance can be found in a poisoning of Cu sites by BHT. At this stage, the results point at the same poisoning effect discussed for the binary catalyst system.

To put these results into a general perspective, and to the best of our knowledge, this is the first time that such a deactivation mechanism is reported for Cu-based liquid-phase hydrogenation reactions.^58^ The purity of the feedstock generally gets less attention in heterogeneous catalysis than in, e.g., homogeneous catalysis. Likely, this is because heterogeneous catalysis mainly focuses on large-volume bulk processes, where purification is costly. In a recent study, Du et al.^59^ reported that the styrene hydrogenation over Pd/Al_2_O_3_ was strongly influenced by the presence of 4-*tert*-butylcatechol (TBC), with a sharp decrease of the reaction rate for concentrations ranging from 15 to 150 ppm. TBC is a free-radical scavenger typically added at the ppm level during transportation and handling. In that case, TBC was a reagent’s additive and it gave rise to a comparable poisoning deactivation as that reported here for BHT on Cu-based catalysts. There is a striking difference; while the work of Du et al.^59^ refers to a supported Pd/Al_2_O_3_ catalyst (0.5 wt % Pd), the effect reported in this study is about catalysts having an enormous concentration of Cu (∼70 wt % for binary catalyst and 60–68 wt % for the commercial). This indirectly proves that these Cu catalysts behave as supported catalysts in the sense that the concentration of active sites is small.

## 4 Concluding Remarks

The hydrogenolysis of γ-butyrolactone over a Cu–ZnO catalyst was found to be dramatically influenced by the solvent’s purity. The effect was preliminarily ascribed to a solvent additive, 2,6-di-*tert*-butyl-4-methylphenol (BHT), present in commercial THF. Such a solvent additive is commercially added as a free-radical scavenger that prevents the formation of peroxides due to health and safety considerations for transportation. The catalyst deactivation is complex to understand, and a preliminary explanation is proposed, namely, BHT-induced poisoning. Though the binary catalyst suffered Cu sintering yielding large particles, this cannot explain the total deactivation. The latter is ascribed to BHT poisoning on the Cu–ZnO interfaces; this explanation is supported by pulsed-N_2_O quantification and control experiments. The BHT effect was also visible when assessing a commercial Cu–ZnO–MgO–Al_2_O_3_ catalyst, though the effect was less severe than for the binary catalyst. This is likely due to the larger surface area and higher active site concentration as compared to those of the binary catalyst. Hence, the commercial catalyst is more tolerant to that impurity. A BHT-induced poisoning was also proposed for the commercial catalyst’s behavior. Generally speaking, the results put forward the need for careful solvent selection and purification in three-phase-catalyzed hydrogenation reactions to achieve optimal performance.

## Supporting Information Available

Description of the chemicals employed for the catalyst synthesis; chemicals employed for the catalytic tests; summary of the reaction conditions; physicochemical properties of the employed solvents; structural properties of the commercial Cu–ZnO–MgO–Al_2_O_3_ catalyst after reaction; calculation of the surface ZnO; process flow diagram of the in-house setup employed for the synthesis of the Cu–Zn hydroxycarbonates; block diagram showing the preparation and activation of the catalyst precursor and final Cu–ZnO catalyst; process flow diagram of the TPR apparatus; process flow diagram of the semibatch autoclave reactor with an advanced reactant feeding system employed for the catalytic tests; XRD patterns of relevant binary catalysts; temperature-programmed reduction of the commercial CuO–ZnO–MgO–Al_2_O_3_ catalyst; concentration profiles corresponding to the experiments reported in the main text (Figure 8); and XPS spectra of the Mg LMM Auger line, as well as of the O 1s and C 1s core level regions (PDF)

## Abbreviations

BDO: 1,4-butanediol
BET: Brunauer–Emmett–Teller
BHT: 2,6-di-*tert*-butyl-4-methylphenol
EPA: USA Environmental Protection Agency
FID: flame-ionization detector
GBL: γ-butyrolactone
GC: gas chromatography
ICP-OES: inductively coupled plasma optical emission spectrometry
JCPDS: Joint Committee on Powder Diffraction Standards Database
MA: maleic anhydride
NFPA: National Fire Prevention Association
SA: succinic anhydride
TCD: thermal conductivity detector
THF: tetrahydrofuran
TPR: temperature-programmed reduction
UNIQUAC: universal quasi-chemical activity coefficient model
XRD: X-ray diffraction
XPS: X-ray photoelectron spectroscopy
