# Correction to “Solvent Additive-Induced Deactivation of the Cu–ZnO(Al_2_O_3_)-Catalyzed γ-Butyrolactone Hydrogenolysis: A Rare Deactivation Process”

**DOI:** 10.1021/acs.iecr.3c00343

**Published:** 2023-02-14

**Authors:** Vanessa Solsona, Silvia Morales-de la Rosa, Oreste De Luca, Harrie Jansma, Bart van der Linden, Petra Rudolf, José M. Campos-Martin, María Emma Borges, Ignacio Melián-Cabrera

The authors regret that Figure 8 contained a typographic error in the original publication. The *x*-axis labels should have been labeled as Time (min) instead of Conversion (%). The discussion in the text is correct and remains valid. However, the wrong labeling of the axes can lead to confusion. Below, an amended Figure 8 can be found. The authors would like to apologize for any inconvenience caused.

**Figure 8:**
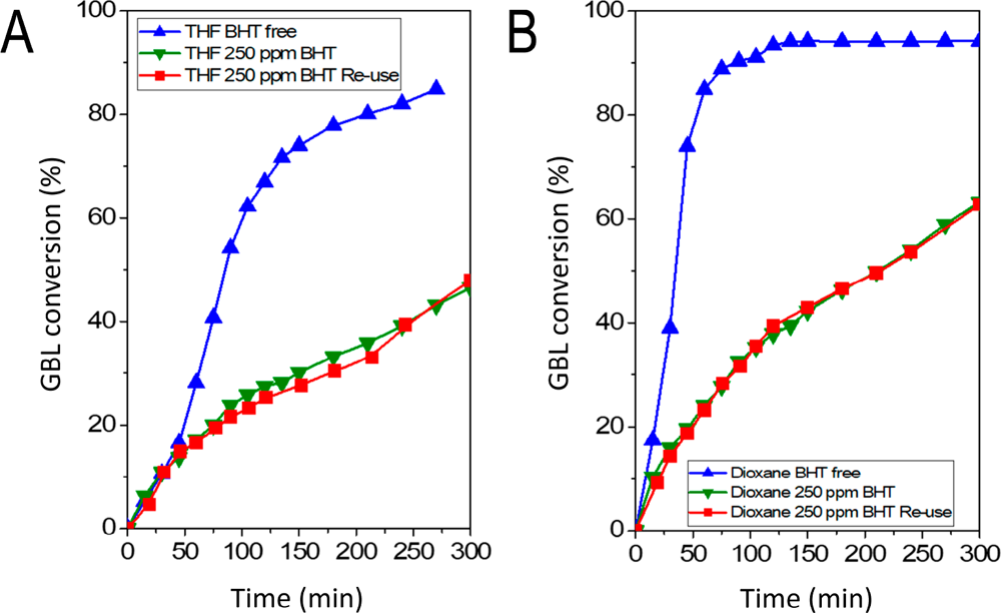
Performance of a commercial Cu-ZnO-MgO-Al_2_O_3_ catalyst in the hydrogenation of γ-butyrolactone using (A) THF as a solvent, with and without BHT as an additive, and (B) using 1,4-dioxane as a solvent, with and without BHT as an additive. For THF, BHT comes as an additive in one of the commercial grades, whereas for 1,4-dioxane, the BHT was added by us to reach the same concentration, 250 ppm. For the experiments containing BHT, the catalyst was reused in a second cycle using fresh reagents after *in situ* reduction. The concentration profiles can be found in Figure S7 (original manuscript). GBL, γ-butyrolactone; THF, tetrahydrofuran; BHT, 2,6-di-*tert*-butyl-4-methylphenol.

