# Supplementary material for: Solvent Additive-Induced Deactivation of the Cu–ZnO(Al2O3)-Catalyzed γ-Butyrolactone Hydrogenolysis: A Rare Deactivation Process
Source: Ind Eng Chem Res. 2021 Oct 27;60(44):15999–6010. doi: 10.1021/acs.iecr.1c04080 (PMC8689444; doi:10.1021/acs.iecr.1c04080)
Supplement: Supplementary file 1 — ie1c04080_si_001.pdf [file ie1c04080_si_001.pdf]

Electronic Supporting Information

Solvent additive-induced deactivation of the Cu-ZnO(Al_2O_3)-catalysed γ-butyrolactone hydrogenolysis: a rare deactivation process

Vanessa Solsona,[1,§] Silvia Morales-de la Rosa,[2,§] Oreste De Luca,[3,§] Harrie Jansma,[1,4] Bart van der Linden,[1,4] Petra Rudolf,[3] José M. Campos-Martin[2,*] María Emma Borges,[5,6] and Ignacio Melián-Cabrera [1,6,*]

1. DelftChemTech, Faculty of Applied Sciences, Delft University of Technology, Julianalaan 136, 2628 BL Delft, The Netherlands.

2. Sustainable Energy and Chemistry Group, Institute of Catalysis and Petrochemistry, CSIC, Marie Curie, 2 Cantoblanco, 28049 Madrid, Spain.

3. Zernike Institute for Advanced Materials, University of Groningen, Nijenborgh 4, Groningen, 9747 AG, The Netherlands.

4. Department of Chemical Engineering, Faculty of Applied Sciences, Delft University of Technology, Van der Maasweg 9, 2629 HZ Delft, The Netherlands.

5. Department of Chemical Engineering, School of Engineering and Technology, University of La Laguna, Avda. Astrofísico Francisco Sánchez, s/n, PO BOX 456, 38200 San Cristóbal de La Laguna, S/C de Tenerife, Spain.

6. Applied Photochemistry and Materials for Energy Group, University of La Laguna, Avda. Astrofísico Francisco Sánchez, s/n, PO BOX 456, 38200 San Cristóbal de La Laguna, S/C de Tenerife, Spain.

* Corresponding authors:

jm.campos@csic.es

ignacio.melian.cabrera@ull.edu.es

ADDITIONAL TABLES AND CALCULATIONS

Table S1. Chemicals used for the synthesis of the Cu-Zn precursors.

Compound	CAS number	Supplier	Purity (%)
$Cu(NO_3)_2{\cdot}3H_2O$	221-838-5	Merck	99.5
$Zn(NO_3)_2{\cdot}6H_2O$	10196-18-6	J.T. Baker	99.0
Na_2CO_3	497-19-8	J.T. Baker	99.5
NaOH	1310-73-2	J.T Baker	99.0

Table S2. Chemicals employed for the binary catalyst testing (Cu-ZnO).

Compound	CAS number	Supplier	Purity (%)
γ-butyrolactone	96-48-0	Aldrich	99.0^+
1,4-Butanediol	110-63-4	Riedel de Haen	≥99.0
Tetrahydrofuran (A)	109-99-9	Riedel de Haen	≥99.9 (a)
Tetrahydrofuran (B)	109-99-9	Aldrich	≥99.9 (b)
1,4-Dioxane	123-91-1	Aldrich	≥99.0

(**a**) contains ~250 mg/kg 2,6-di-*tert*-butyl-4-methylphenol (BHT) as stabilizer
(**b**) Ultrapure, BHT-free.

Table S3. Chemicals employed for the commercial catalyst testing (Cu-ZnO-MgO-Al_2O_3).

Compound	CAS number	Supplier	Purity (%)
γ-butyrolactone	96-48-0	Aldrich	99.0+
1,4-Butanediol	110-63-4	Aldrich	99.0+
Tetrahydrofuran (A)	109-99-9	Aldrich	≥99.9 (a)
Tetrahydrofuran (B)	109-99-9	Aldrich	≥99.9 (b)
1,4-Dioxane	123-91-1	Aldrich	≥99.8
BHT	128-37-0	Aldrich	≥99.0

(**a**) contains ~250 mg/kg 2,6-di-*tert*-butyl-4-methylphenol (BHT) as stabilizer.
(**b**) Ultrapure, BHT-free.

Table S4. Experimental conditions for the semi-batch reactor experiments.

**Conditions**	**Quantity, observation**
Temperature, T (°C)	180
Initial concentration, C_o (mol/l)	0.325
Total pressure, p (MPa)	5
Volume of the reaction mixture, V_o (ml)	200 or 461
Catalyst weight, m_{cat} (g)	3 or 6.75 (oxide)
Particle size, d_p (μm)	<100
Reactor stirrer speed, $n_{stirrer}$ (rpm)	1500
Solvents employed	1,4-Dioxane (≥99.0, Aldrich)<br>1,4-Dioxane (≥99.8, Aldrich) containing BHT [(a)]<br>THF type A: Tetrahydrofuran containing BHT [(b)]<br>THF type B: Tetrahydrofuran, ultrapure, BHT free [(c)]

(**a**) The BHT (2,6-di-*tert*-butyl-4-methylphenol) was added by us to reach 250 ppm.

(**b**) Commercial product; it contains *ca.* 250 ppm BHT as stabilizer.

(**c**) This is an ultra-pure HPLC-grade BHT-free THF.

Table S5. Physico-chemical properties of the employed solvents.[(a)]

**Solvent**	**ρ (g/cm³)**	**μ (mPa s)**
1,4-Dioxane	1.033	1.177
Tetrahydrofuran	0.888	0.456

(**a**) D.R. Lide (Editor), Handbook of Chemistry and Physics, 89th Ed. CRC Press, 2008.

Table S6. Structural properties of the commercial Cu-ZnO-MgO-Al_2O_3 catalyst after various reactions.

**Catalyst**	**Properties**	**Cu^{XRD} (nm)** [(a)]
Commercial	Reduced, after reaction, THF type A (BHT)	5.8
Commercial	Reduced, after reaction, dioxane (BHT)	6.5
Commercial	Reduced, after reaction, THF type B (pure)	7.7
Commercial	Reduced, after reaction, dioxane (pure)	6.3

(**a**) From the XRD patterns in Figure 7.

Calculation of the surface ZnO

$$ZnO_{sites}^{surface} \cong 3\ g\ cat\ \times \left(10.2\ \cdot 10^{-2}\ \frac{mmol\ N_2O}{g\ cat}\right) \times \frac{2\ Cu}{1\ N_2O} \times \frac{3\ mol\ Zn}{7\ mol\ Cu} = 262\ \mu mol\ ZnO$$

In this calculation, we have assumed that the Cu:Zn ratio remains equal on surface, which is a good assumption since these catalysts are bulky. In grey is the chemisorption derived data.

ADDITIONAL FIGURES

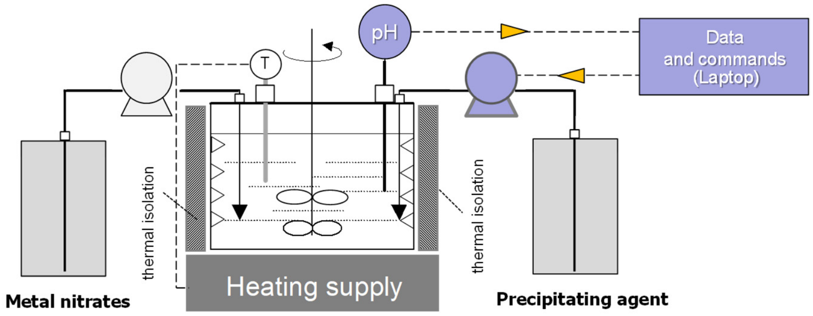

Figure S1. Process flow diagram of the tailor-made set-up employed for the synthesis of the Cu-Zn hydroxycarbonate, where the pH of the suspension was automatically controlled. The pH controlling system consisted of pH electrode, pump, data-transfer interface and laptop-controlled Labview® software. An external peristaltic pump was used to add the metal nitrates.

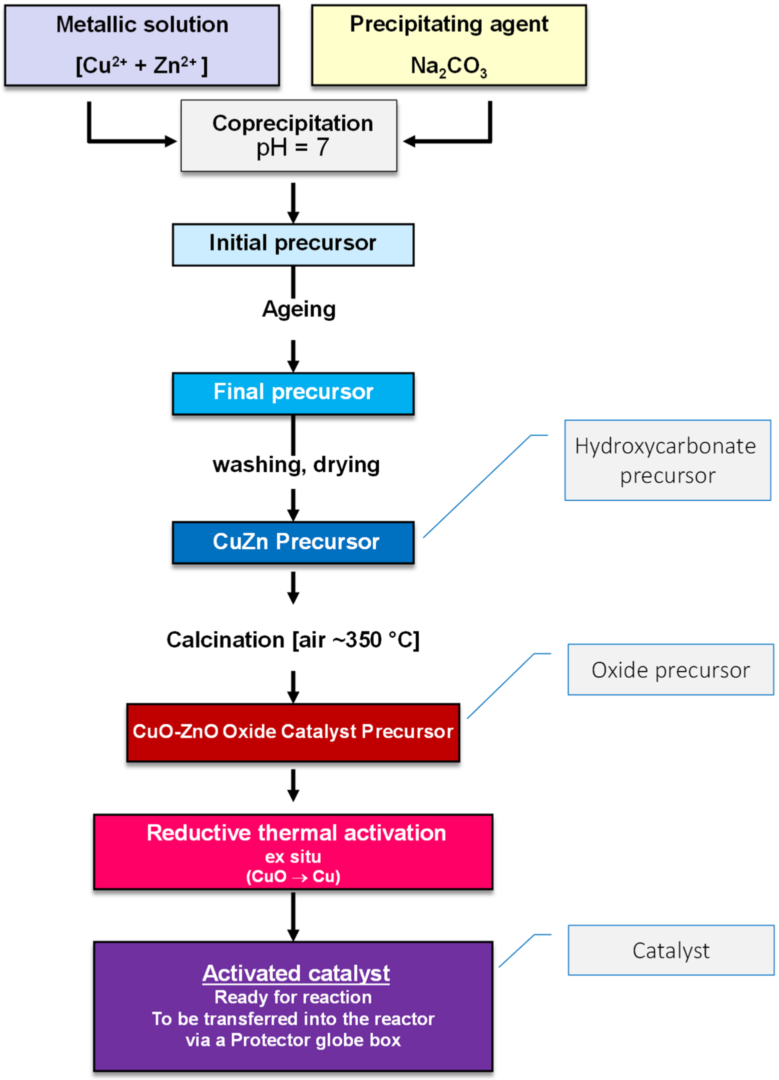

Figure S2. Block diagram showing the preparation and activation of the catalyst precursor and final Cu-ZnO catalyst, starting from a metallic solution up to the active catalyst before the reaction.

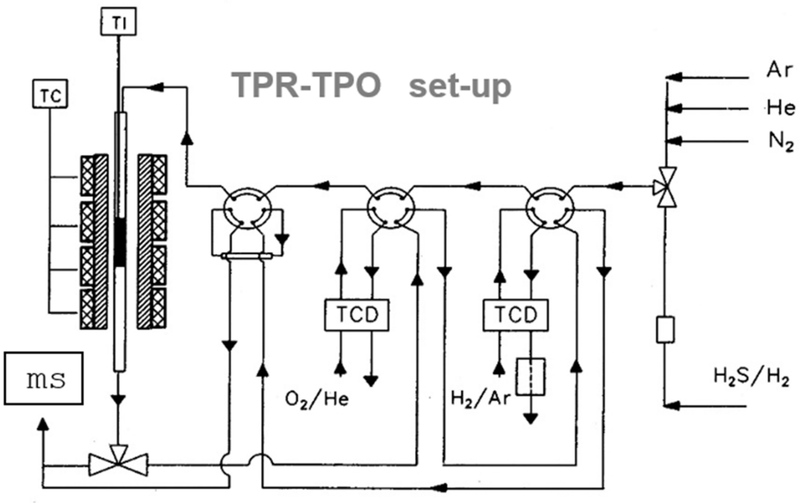

Figure S3. Process flow diagram of the apparatus for temperature-programmed reduction and temperature-programmed oxidation (**TPR**-**TPO**) used in this research.

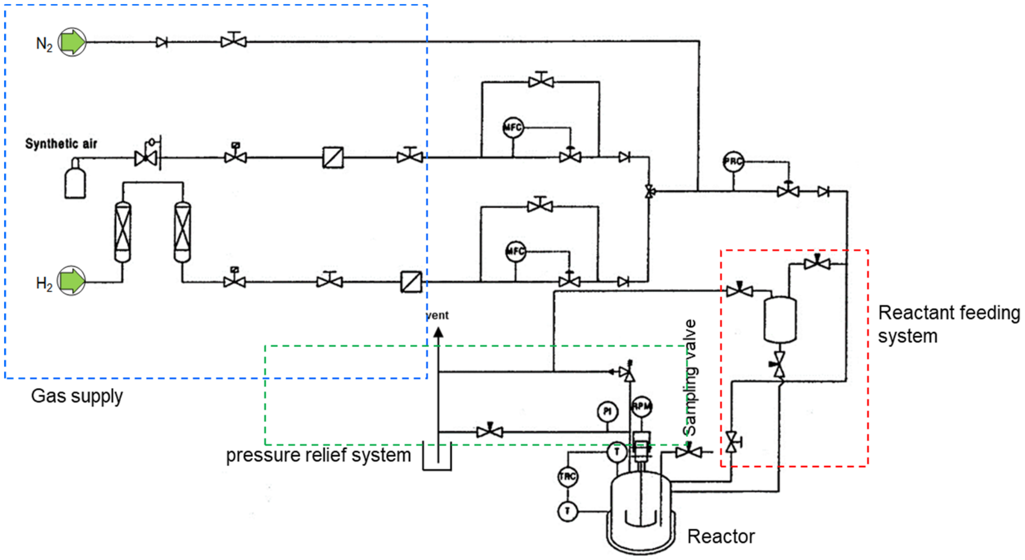

Figure S4. Process flow diagram of the state-of-the-art semi-batch autoclave reactor, with an advanced reactant feeding system. The reactant (γ-butyrolactone) was fed into the reactor through a pressurised vessel once the reaction temperature was reached.

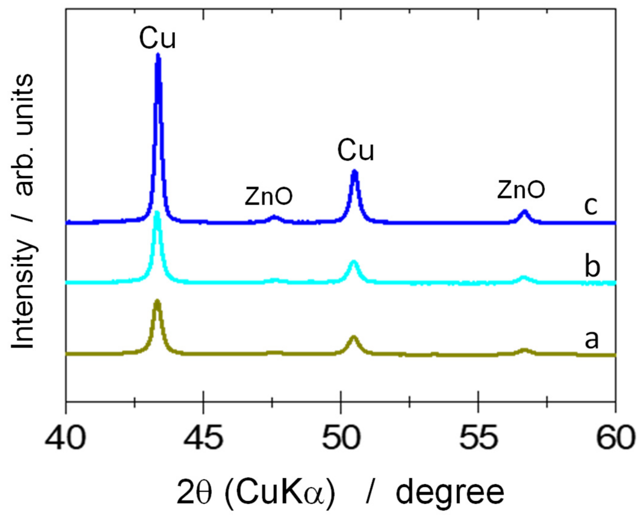

Figure S5. XRD patterns for the binary Cu-ZnO catalyst: a) after reduction; b) reduced, after reaction in pure dioxane; c) Reduced, after reaction using THF type A.

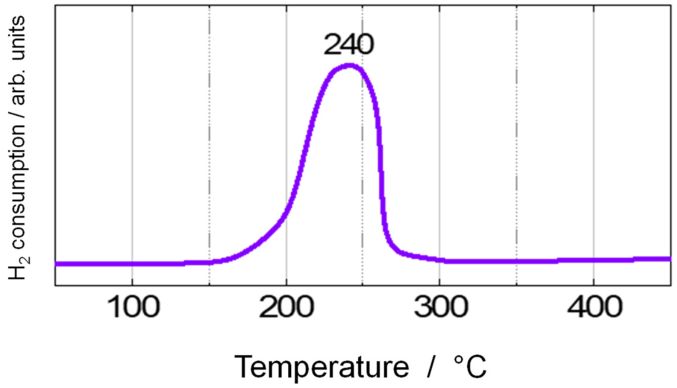

Figure S6. Temperature-programmed reduction of the commercial CuO-ZnO-MgO-Al_2O_3 catalyst.

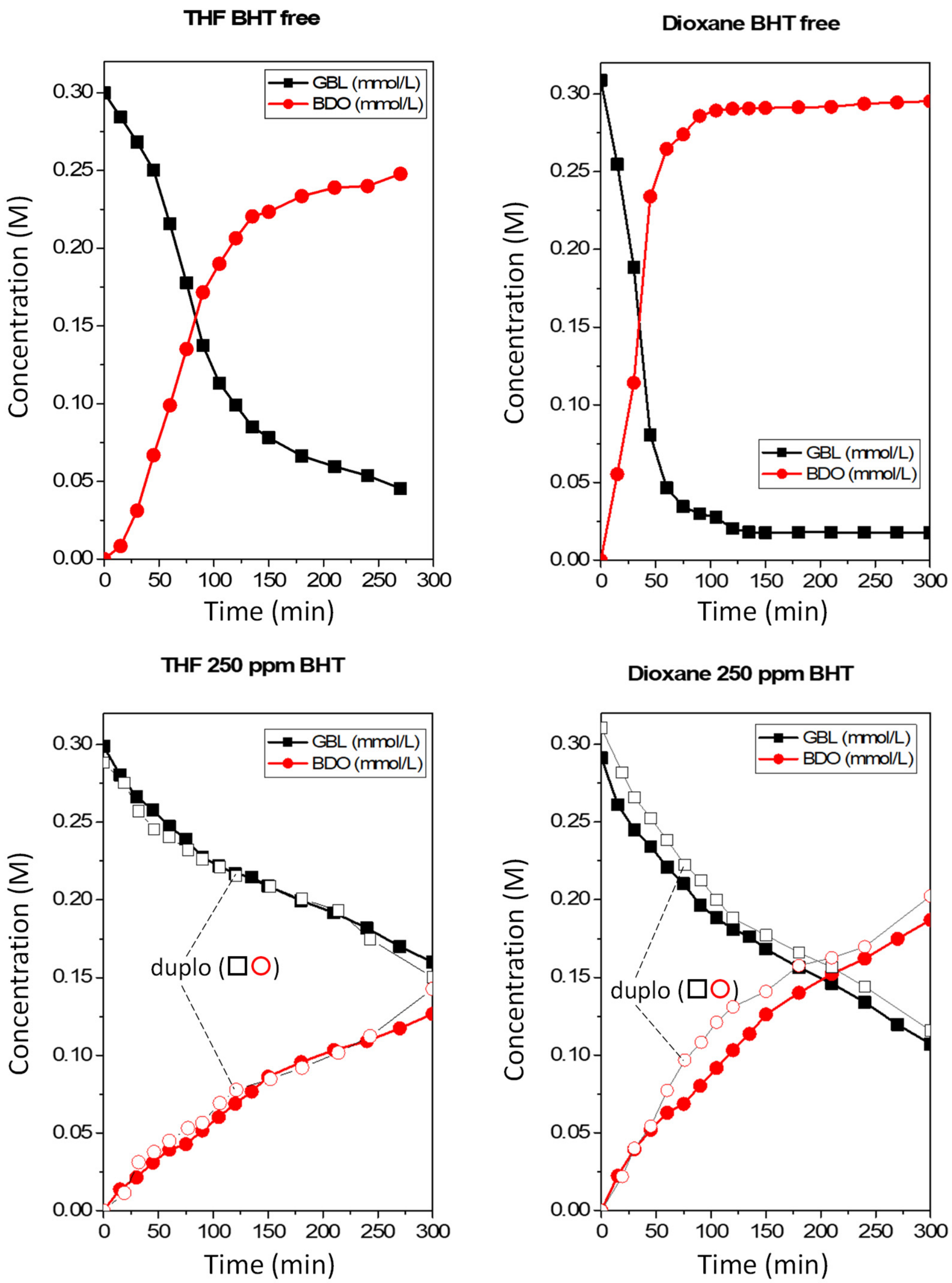

Figure S7. Concentration profiles corresponding to the experiments reported in Figure 8. GBL= γ-butyrolactone; BDO = 1,4-Butanediol.

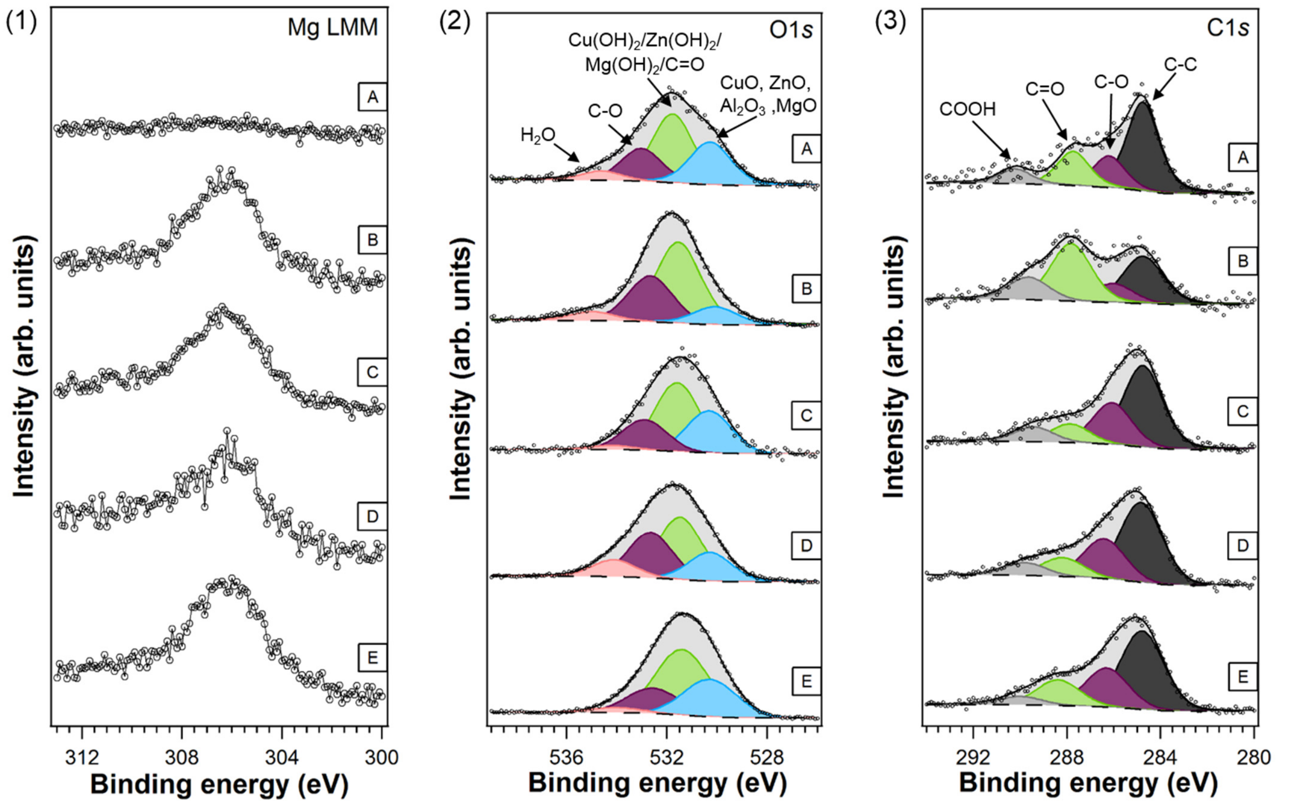

Figure S8. CuO-ZnO-MgO-Al_2O_3 system. XPS spectra of the (**1**) Mg LMM Auger line, (**2**) O1s and (**3**) C1s core level regions before and after different reduction treatments. Materials: (**A**) fresh commercial unreduced catalyst; (**B**) after-reaction, pure THF; (**C**) after-reaction, pure dioxane; (**D**) after-reaction, BHT-containing THF and (**E**) after-reaction, BHT-containing dioxane. THF = Tetrahydrofuran; BHT = 2,6-di-*tert*-butyl-4-methylphenol.
